# Comprehensive Analysis of ^13^C_6_ Glucose Fate in the Hypoxia-Tolerant Blind Mole Rat Skin Fibroblasts

**DOI:** 10.3390/metabo11110734

**Published:** 2021-10-27

**Authors:** Dmitry Miskevich, Anastasia Chaban, Maria Dronina, Ifat Abramovich, Eyal Gottlieb, Imad Shams

**Affiliations:** 1 Department of Evolutionary and Environmental Biology, Faculty of Natural Sciences, University of Haifa, Haifa 3498838, Israel; anastasia@chaban.su; 2 Institute of Evolution, University of Haifa, Haifa 3498838, Israel; dronina.maria@gmail.com; 3 Technion Faculty of Medicine, Haifa 3525433, Israel; ifat.a.g@gmail.com (I.A.); e.gottlieb@technion.ac.il (E.G.)

**Keywords:** metabolome, hypoxia, proline cycle, GSH, adaptation, bioenergetics, *Spalax*, glucose

## Abstract

The bioenergetics of the vast majority of terrestrial mammals evolved to consuming glucose (Glc) for energy production under regular atmosphere (about 21% oxygen). However, some vertebrate species, such as aquatic turtles, seals, naked mole rat, and blind mole rat, *Spalax*, have adjusted their homeostasis to continuous function under severe hypoxic environment. The exploration of hypoxia-tolerant species metabolic strategies provides a better understanding of the adaptation to hypoxia. In this study, we compared Glc homeostasis in primary *Spalax* and rat skin cells under normoxic and hypoxic conditions. We used the targeted-metabolomics approach, utilizing liquid chromatography and mass spectrometry (LC-MS) to track the fate of heavy Glc carbons (^13^C_6_ Glc), as well as other methodologies to assist the interpretation of the metabolic landscape, such as bioenergetics profiling, Western blotting, and gene expression analysis. The metabolic profile was recorded under steady-state (after 24 h) of the experiment. Glc-originated carbons were unequally distributed between the cytosolic and mitochondrial domains in *Spalax* cells compared to the rat. The cytosolic domain is dominant apparently due to the hypoxia-inducible factor-1 alpha (HIF-1α) mastering, since its level is higher under normoxia and hypoxia in *Spalax* cells. Consumed Glc in *Spalax* cells is utilized for the pentose phosphate pathway maintaining the NADPH pool, and is finally harbored as glutathione (GSH) and UDP-GlcNAc. The cytosolic domain in *Spalax* cells works in the semi-uncoupled mode that limits the consumed Glc-derived carbons flux to the tricarboxylic acid (TCA) cycle and reduces pyruvate delivery; however, it maintains the NAD^+^ pool via lactate dehydrogenase upregulation. Both normoxic and hypoxic mitochondrial homeostasis of Glc-originated carbons in *Spalax* are characterized by their massive cataplerotic flux along with the axis αKG→Glu→Pro→hydroxyproline (HPro). The product of collagen degradation, HPro, as well as free Pro are apparently involved in the bioenergetics of *Spalax* under both normoxia and hypoxia. The upregulation of 2-hydroxyglutarate production detected in *Spalax* cells may be involved in modulating the levels of HIF-1α. Collectively, these data suggest that *Spalax* cells utilize similar metabolic frame for both normoxia and hypoxia, where glucose metabolism is switched from oxidative pathways (conversion of pyruvate to Acetyl-CoA and further TCA cycle processes) to (i) pentose phosphate pathway, (ii) lactate production, and (iii) cataplerotic pathways leading to hexosamine, GSH, and HPro production.

## 1. Introduction

Evolution created myriads of species well adapted to their environments. The vast majority of terrestrial animals evolved to inhale about 21% oxygen. The metabolism of these animals is fine-tuned to process glucose along the glycolytic axis and to ‘burn’ glucose (Glc) derived pyruvate in the oxidative phosphorylation process (OXPHOS) to generate energy. The metabolic pathways of many organisms are well documented both on the cellular and organismal levels. However, there are vertebrate species that do not necessarily follow the ‘full-oxygen supply addiction’ rule, such as the naked mole-rat [1], some species of turtles [2], seals, and whales [3], whose metabolism is well adapted to low oxygen availability. One of these outstanding group of animals is the blind mole rat of *Spalax ehrenbergi* complex (*spalacidae*) (hereafter, *Spalax*). *Spalax* is an underground, solitary, long-living mammal inhabiting sealed underground burrows under extreme fluctuations in O_2_/CO_2_ levels. *Spalax* evolved during millions of years in low-oxygen atmosphere and tolerates severe hypoxia (3% under laboratory conditions, and down to ~7% oxygen and ~6% carbon dioxide recorded in natural habitat [4]). *Spalax* extraordinary longevity (up to 20 years) [5], oxidative stress tolerance [6] and cancer resistance [7] can probably be attributed to their ability to bear hypoxia. Hypoxia is challenging for mammalian cells for two main reasons: oxygen availability is crucial for mitochondrial ATP production where it is used as an electron acceptor, as well as for maintenance of oxygen-dependent cellular homeostasis (signaling, maintaining mitochondrial membrane potential, fatty acids desaturation, hydroxylation processes, etc. [8,9,10,11,12]). A main respiration-related nutrient is glucose. The steady-state respiration is fueled by complete oxidation of Glc, whereas hypoxic mode is based on its anaerobic fermentation. The anaerobic Glc fermentation is much less effective than OXPHOS and imposes additional challenges to the cell, such as lactic acidosis [13]. The ability of *Spalax* to tolerate low oxygen atmosphere for extended periods suggests specific adaptations that motivated us to explore the metabolic fate of Glc in the cells with the lowest physioxic levels in the body, skin tissues. The aim of the present study was to compare the metabolism of Glc in the primary cells of two species similar by mass, size, taxonomy, but different in their adaptations: wild *Spalax* and white laboratory rat (*Rattus norvegicus*). For this purpose, wild-captured newborn *Spalax* and purchased newborn rats were used for the isolation of primary dermal fibroblasts. The fate of Glc-derived carbons in primary *Spalax* and rat cells was explored and compared under resting mode as well as under hypoxic conditions. 

## 2. Results

The distributions of heavy glucose carbons through the metabolic landscape after one day of the experiment demonstrated a complete ^13^C enrichment picture. These metabolites were designated in the study as the metabolite’s name followed by ‘M + n’ (‘M’ stands for molecular mass; n indicates the number of heavy carbons ^13^C in the metabolite). The mass isotopologue distribution (MID) for most important metabolites of Glc is presented in Supplementary Figure S1.

**Figure 1:**
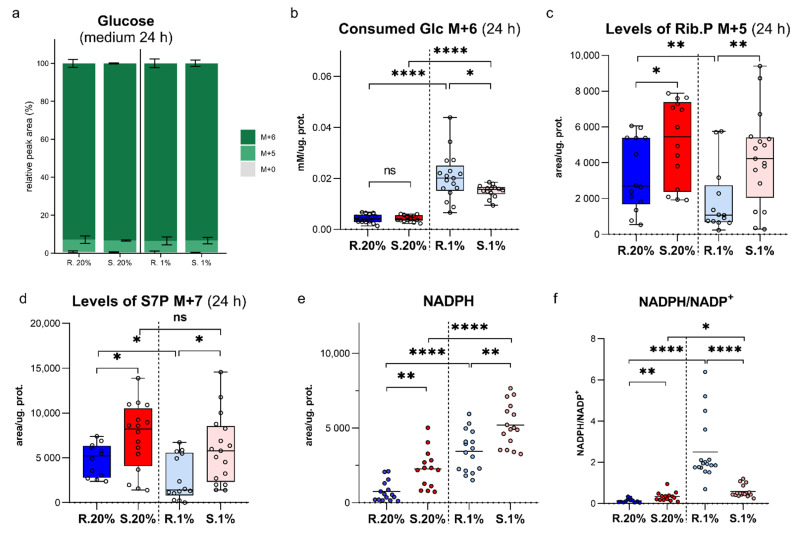
Mass isotopologue distribution (MID) for Glc in the culture media and consumption of Glc M + 6 by growing *Spalax* and rat cells. (**a**), Distribution of heavy carbons in culture media after 24 h; (**b**), the consumption of glucose by *Spalax* and rat cells under normoxia (20%) and hypoxia (1%); characteristics of the pentose phosphates pathway in normoxic and hypoxic *Spalax* and rat cells: (**c**), ribose phosphates (Rib.5P M + 5); (**d**), sedoheptulose-7-phosphate (S7P M + 7); (**e**), NADPH; (**f**), ratio of NADPH/NADP in *Spalax* and rat cells after 24 h of growing under normoxia and hypoxia. S.20%, R. 20%, S.1%, R1% represent *Spalax* (S) and rat (R) cells exposed to an atmosphere containing 20% or 1% O_2_, respectively; ns, (nonsignificant) *p* > 0.05; * *p* < 0.05; ** *p* < 0.01; **** *p* < 0.0001, error bars represent standard deviation of 6 or more biological repeats. Every point on the chart represents one technical repeat.

Glc M + 6 is a dominant mass isotopologue (MI) in the culture medium (>93% of Glc) (Figure 1a) and further, the Glc homeostasis is discussed as transformations of consumed heavy Glc M + 6 carbons. There was no difference in Glc consumption between *Spalax* and rat cells under normoxia (Figure 1b); however, the intracellular distribution of consumed carbons was remarkably different between the two species. Low oxygen conditions caused the upregulation of Glc M + 6 uptake by both cells compared to normoxia; however, higher values of Glc consumption were recorded for rat cells (Figure 1b).

### 2.1. An Upregulated Pentose Phosphate Pathway (PPP) in Spalax Cells Provides Higher Levels of NADPH

PPP generates pentoses for nucleotide production and maintains an intracellular pool of reduced NADPH. The levels of PPP oxidative products, ribose phosphates (Rib.5P) and sedoheptulose-7-phosphate (S7P), were significantly higher in *Spalax* cells compared to the rat under normoxia (Figure 1c,d), in line with elevated levels of NADPH (Figure 1e). The value of the predominantly cytosolic NADPH/NADP^+^ ratio indicates that *Spalax* cells are more reduced under normoxia (Figure 1f). The levels of PPP-derived ribose phosphates in hypoxic *Spalax* cells were similar to those under normoxia and remained high compared to hypoxic rat cells; on the other hand, the levels of Rib.5P M + 5 and S7P M + 7 were decreased in rat cells under hypoxia (Figure 1c,d). The levels of NADPH were increased in both hypoxic cells. These observations provided evidence of a slowdown of reactions that utilize NADPH. Interestingly, the decrease of NADPH-using processes is more pronounced in hypoxic rat cells (Figure 1e,f).

### 2.2. Spalax Cells Branched Much More Consumed Glucose to Hexosamine Biosynthetic Pathway (HBP), but Not to Hyaluronic Acid Production

**Figure 2:**
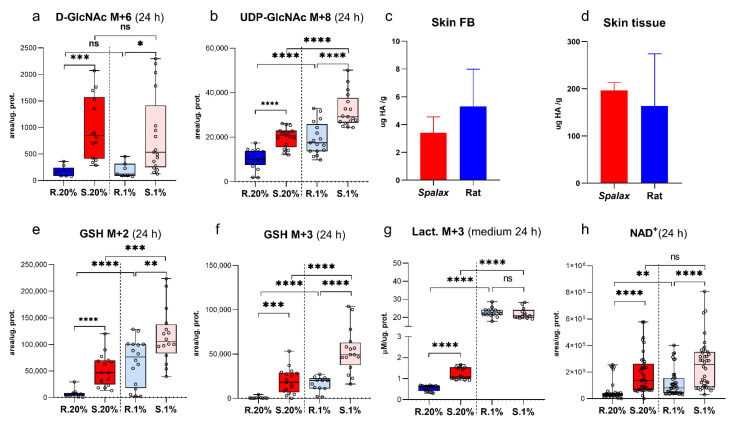
Characteristics of hexose amines pathway and glycolysis. Levels of (**a**), d-GlcNAc M + 6; (**b**), UDP-GlcNAc M + 8; (**e**), GSH M + 2; (**f**), GSH M + 3; (**h**), NAD^+^ in *Spalax* and the rat skin cells extracts; concentrations of hyaluronic acid in: (**c**), *Spalax* and rat skin fibroblasts; (**d**), *Spalax* and rat skin tissues; (**g**), concentrations of Lact M + 3 secreted outside by the cells after 24 h of the experiment. S.20%, R. 20%, S.1%, and R1% represent *Spalax* (S) and rat (R) cells exposed to an atmosphere containing 20% or 1% O_2_, respectively; ns, (non-significant) *p* > 0.05; * *p* < 0.05; ** *p* < 0.01; *** *p* < 0.001; **** *p* < 0.0001, error bars represent standard deviation of 6 or more biological repeats. Every point on the chart represents one technical repeat.

A robust flux of heavy carbons (increased levels of the N-acetylglucosamine (GlcNAc) M + 6 and Uridine Diphosphate N-acetylglucosamine (UDP-GlcNAc M + 8) was observed along with the HBP in *Spalax* cells (Figure 2a,b). In contrast with our previous assumption and other published data [14], *Spalax* cells did not direct glucose to hyaluronic acid (HA) production (Figure 2c,d). The only known way for glucose in HBP besides HA path is UDP-GlcNAc production together with the following glycosylation of proteins [14]. The extreme sensitivity of *Spalax* cells to treatment with tunicamycin (Figure S2(Aa)), an inhibitor of GlcNAc phosphotransferase (the key enzyme of glycosylation) [15] in complex with increased production of UDP-GlcNAc suggests an upregulation and critical role of glycosylation in the *Spalax* cell viability.

The levels of UDP-GlcNAc M + 6 remained elevated in hypoxic *Spalax* cells compared to rat, but unchanged relative to its normoxic values (Figure 2a). The combination of UDP-GlcNAc M + 6 with Ac-CoA M + 2 -UDP-GlcNAc M + 8 increased in both types of cells under hypoxia, with the highest levels of GlcNAc M + 8 observed in *Spalax* cells (Figure 2b). This indicates an increased back flux of mitochondrial Ac-CoA M + 2 to the cytosolic production of UDP-GlcNAc in hypoxic *Spalax* cells.

### 2.3. Spalax Cells Massively Redirect Glucose Carbons to the Synthesis of the Tripeptide Glu-Cys-Gly, Glutathione (GSH)

Notably, a massive redirection of glucose carbons to the GSH biosynthesis was observed in normoxic *Spalax* cells. The unpredictable combinations of both labeled Glc-derived and non-labeled Gly, Ser, Cys (not detected by the method) and Glu resulted in the appearance of appropriate MIs of GSH presented in Figure 2e,f and Figure S2B. The levels of dominant MIs of GSH (Figure S1e and Figure 2e,f) as well as the sum of all detected MI of GSH (Figure S2(Bg)) were substantially higher in *Spalax* cells. Interestingly, the levels of the oxidized form of GSH (GSSG) were significantly higher in rat cells under hypoxia and normoxia (Figure S2(Bh)). Taking in account the specific intracellular redox-maintaining function of GSH [16], this suggests a lower rate of oxidative processes in *Spalax* cells. Hypoxia induces GSH production for both cell types; nonetheless, the levels of all major measured MI of GSH in *Spalax* cells, as well as their sum, were significantly higher as compared to the rat, with notably higher oxidation rate of GSH to GSSG in hypoxic rat cells (Figure 2e,f and Figure S2(Bg,Bh)).

### 2.4. Upregulated Lactate Production in Spalax Cells Maintains NAD^+^ Pool for Glycolytic Machinery and Rejects Glucose-Derived Carbons from Downstream Processing in the TCA Cycle

Pyruvate is mainly generated via the Embden–Meyerhof pathway that has several possible fates, and mainly these two: pyruvate enters the mitochondria and is processed in the TCA cycle, or is interconverted by alanine transaminase (ALT) to Ala, or by lactate dehydrogenase (LDH) to Lact [17]. Resting (normoxic) *Spalax* cells produce and secrete more Lact M + 3 than rat (Figure S1i and Figure 2g). The cells secrete lactate to prevent lactic acidosis development [18]; thus, the higher Lact M + 3 concentration secreted outside is due to active LDH inside the cell. The LDH reaction of interconversion of Pyr to Lact is redox-coupled and influences the redox state of the cell via NAD^+^ regeneration and maintains the NAD^+^ pool for anaerobic glycolysis [17,18]. Seemingly, even resting *Spalax* cells require LDH-derived NAD^+^ for glyceraldehyde 3-phosphate dehydrogenase for more effective processing of trioses. Probably, because of activated LDH, the redox state of *Spalax* cells is shifted to a more oxidized state than in the rat (Figure 2h and Figure S2(Ab)). Nonetheless, LDH activation positively maintains the intracellular NAD^+^ pool, while the massive secretion of lactate by *Spalax* cells excludes glucose-derived carbons from further metabolism. Interestingly, the conversion of pyruvate to alanine as well as its secretion outside the cell in normoxic *Spalax* cells are dramatically diminished compared to the rat cells (Figures S1h and S2(Ac,Ad)). Hypoxia induced an enormous increase of Lact M + 3 production and secretion in both species’ cells at similar levels (Figure 2g and Figure S1i). Interestingly, hypoxic stress changes redox state manifested as a decreased ratio of NADH over NAD^+^ in rat cells, while this value was not altered in *Spalax* (Figure S2(Ab)). Along with increasing Lact M + 3 production, the conversion of Pyr M + 3 to Ala M + 3 was also raised under hypoxia in both cells, whereas these values remained lower in *Spalax* cells (Figures S1h and S2(Ac,Ad)).

### 2.5. Spalax Cells Metabolized Less Glc-Originated Pyruvate Entering via Pyruvate Dehydrogenase Complex (PDC) Than the Rat Cells, While Pyruvate Carboxylase (PC)-Mediated Anaplerosis Is More Pronounced in Spalax 

**Figure 3:**
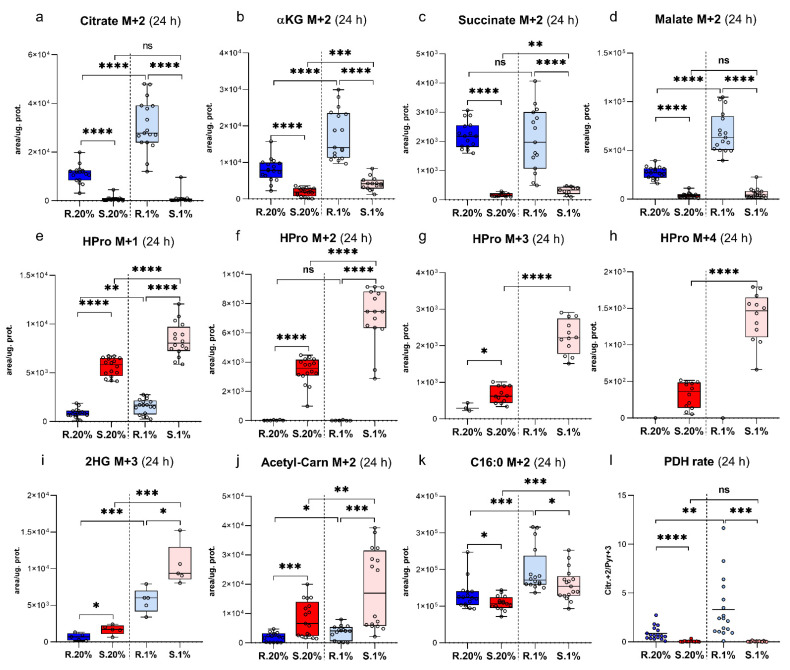
Pyruvate delivery in the TCA cycle via PDH under normoxia; diversion of glucose-derived carbons flux to HPro production; PC-processed carbons to de-novo biosynthesis of 2HG + 3; and fate of Acetyl-CoA(Ac-CoA). (**a**), Intracellular levels of Citr M + 2; (**b**), αKG M + 2; (**c**), Succinate M + 2; (**d**), Malate M + 2; (**e**–**h**), intracellular levels of HPro M + 1; M + 2; M + 3; M + 4; (**i**), intracellular levels of 2HG; (**j**), Acetyl-Carnitine M + 2; (**k**), C16:0 M + 2; (**l**), PDH rate (calculated as ratio of Citr M + 2 over Pyr M + 3 in *Spalax* and rat cells under normoxia and hypoxia. S.20%, R. 20%, S.1%, and R1% represent *Spalax* (S) and rat (R) cells exposed to an atmosphere containing 20% or 1% O_2_, respectively; ns, (nonsignificant) *p* > 0.05; * *p* < 0.05; ** *p* < 0.01; *** *p* < 0.001; **** *p* < 0.0001, error bars represent standard deviation of 6 or more biological repeats. Every point on the chart represents one technical repeat.

The indicated PDH rate for normoxic *Spalax* cells is significantly lower than that in rat cells (Figure 3l), and correspondingly, citrate (Citr) M + 2 levels and fractions in rat cells are bigger than those of *Spalax* (Figure 3a).

The alternative processing of Pyr M + 3, pyruvate anaplerosis, occurs via PC and serves as the way of compensation of the anabolic metabolites leakages [19,20]. In this case, Pyr M + 3 converts into OAA M + 3 (not detected by the present methodology) and further appears as Mal M + 3; Asp M + 3; Asn M + 3. Seemingly, pyruvate anaplerosis that occurs via pyruvate carboxylase is more essential for normoxic *Spalax* cells. The fractions of Mal M + 3, Asp M + 3, and Asn M + 3 are bigger in *Spalax* cells, which indicate the Pyr M + 3 flux via PC. Interestingly, the PC-processed carbons in normoxic *Spalax* cells are diverted to Asn M + 3 production, not to Mal M + 3, as it was observed in rat cells (Figures S1m,s,t, S3B and S5Ac). Alternatively, pyruvate anaplerosis can be detected as the levels of Citr M + 5 (as a result of combination of OAA M + 3 and Ac-CoA M + 2 [21]), which were increased in normoxic *Spalax* cells (Figure S4). The indicated lower PDH rate in *Spalax* hypoxic cells was unchanged compared to normoxic values and stayed significantly lower than that in rat cells (Figure 3l). Remarkably, both Citr M + 2 levels and calculated PDH rate were significantly increased in rat cells under hypoxia (Figure 3a,l).

### 2.6. Succinate Is Essential for Hypoxic Spalax Cells

Notably, hypoxic levels of Succ M + 1, M + 2, M + 3 in *Spalax* hypoxic cells were slightly increased compared to normoxia levels, while the corresponding levels of the downstream TCA cycle’s metabolite—Mal M + 1, M + 2, M + 3—were almost unchanged (Figure S3).

### 2.7. A Large Part of Glc-Originated Carbons Was Diverted to the Metabolism along with the Axis αKG→Glu→Pro→HPro Instead of Processing in the TCA Cycle Reactions

Paradoxically, only a small fraction of Glc-derived carbons was processed through TCA cycle reactions after their conversion to αKG (Figure 3a–d and Figure S3). A significant part of all detected heavy carbons was rejected from the TCA cycle metabolism in both normoxic and hypoxic *Spalax* cells and subjected to a parallel with the TCA cycle metabolism after being tunneled via the αKG-leakage point (Figure 3e–i and Figure S4). These findings were evidenced by the detection of high heavy HPro levels secreted by *Spalax* cells to the growth medium (Figure S2(Ae–Ah)).

### 2.8. Spalax Cells Possess Upregulated Flux of PC-Metabolized Glc Carbons into 2 Hydroxyglutarate (2HG)

PC-metabolized Glc-derived carbons were processed to αKG and further to 2HG (2HG M + 3; +4) in normoxic *Spalax* cells (Figure 3i; Figure S2(Aj,Ak)). Both fractions, 2HG M + 3 and 2HG M + 4, in MID for 2HG are almost equal to 2HG M + 2 in *Spalax* cells, while in rat cells, 2HG M + 2 is dominant over both 2HG M + 3 and 2HG M + 5 (Figure S1p). This indicates an upregulated flux of PC-metabolized carbons into 2HG in normoxic *Spalax* cells. Alongside with the indicated increase of PDH-mediated pyruvate delivery in hypoxic rat cells, a rising of PC pyruvate processing (evidenced as M + 3 MI) was observed (Figure 3g,i). Similar to the observed fraction in normoxic *Spalax* cells, a bigger fraction of Glc-derived carbons branched away from the TCA cycle reactions (Figure S3Ae, OGDH rate) and processed to Glu (Figure S3Af, GDH rate) in rat cells under hypoxia. 

### 2.9. The Newly-Produced Ac-CoA Forwards to Acetyl Carnitine (ALCAR) Synthesis More Than to De-Novo Fatty Acids Production in Spalax Cells

As it was observed in normoxia, *Spalax* cells more readily metabolize Pyr M + 3-derived Ac-CoA M + 2 into ALCAR M + 2 instead of its processing into Citr M + 2 or fatty acid (Figure 3a,j,k). Both cell types amplified the redirection of Glc-originated Ac-CoA M + 2 to the ALCAR M + 2 synthesis and de-novo fatty acids production under hypoxia compared to normoxic conditions (Figure 3j,k). Seemingly, the ALCAR M + 2 production is a more essential strategy for Ac-CoA storage for *Spalax* cells while the production of the fatty acid was more evident in rat cells.

### 2.10. Hypoxic Spalax Cells Significantly Amplified the Harboring of Glc-Derived Carbons That Entered to Mitochondria as HPro 

Regardless of the metabolic route that was used for metabolism, all heavy Glc-carbons detected in *Spalax* cells were observed as HPro M + 0, +1, +2, +3, +4, or heavy metabolites along to the direction from αKG to its terminal point-HPro (Figures S2(Aa–Ad) and S4). 

### 2.11. Spalax Cells Express Higher Levels of Hypoxia- Inducible Factor-1a (HIF-1α) under Normoxia and Hypoxia Compared to the Rat Cells

**Figure 4:**
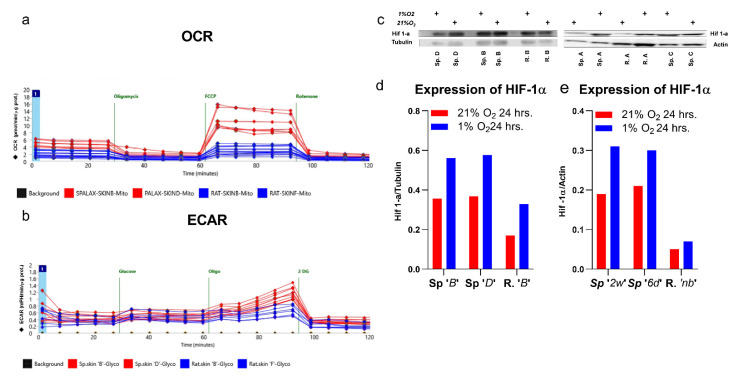
Oxygen consumption rate (OCR) (**a**) and extracellular acidification rate (ECAR) (**b**) in Spalax and rat cells estimated using the Seahorse platform, and expression of HIF-1α in Spalax and the rat skin cells under normoxia and hypoxia after 24 h of the study. Western blot (WB) of HIF-1α for Spalax and rat skin FB using antibodies HIF-1α (28b):sc-13515 (Santa Cruz) (**c**); (**d**,**e**) densitometrical quantification of HIF-1α expression normalized to tubulin and actin, respectively. SKNB, skin newborn ‘B’; SKND, skin newborn ‘D’; RAT-SKINB, rat skin ‘B’; RAT-SKINF, rat skin ‘F’.

The expression of the HIF-1α in *Spalax* cells was significantly higher under normoxia compared to that in the rat cells. Its values were further upregulated under low oxygen atmosphere in both types of cells, but more intensively in *Spalax* (Figure 4c,d). Notably, a similar frame for HIF-1α expression was previously detected in the liver transcriptome (Figure S5(Ba)).

### 2.12. Estimation of the Mitochondrial Aerobic Metabolism (MAM) and the Glycolytic Function (GF) in Spalax and Rat Cells Using the Seahorse Platform

The Seahorse plate-based technology allows to compare the aerobic glycolysis of both cell types using the extracellular acidification rate (ECAR) of the culture medium (Figure 4b).

The glycolysis in *Spalax* cells is higher compared to the rat cells. The ATP synthase inhibition by oligomycin switches Glc flow from mitochondrial OXPHOS, leading to increased Lact production and corresponds to the medium acidification [22], indicating and confirming a shift from OXPHOS to substrate phosphorylation. As it was clearly observed, *Spalax* cells employ stress-induced substrate phosphorylation much more effectively compared to the rat cells. To explore and compare MAM in *Spalax* and rat cells, the XF Mito Stress assay was used (Figure 4a). Paradoxically, the MAM in *Spalax* cells is more oxidative that in the rat; values of basal respiration, ATP production, maximal respiration, and spare capacity in *Spalax* cells are higher as compared to the rat cells. The OCR values in *Spalax* cells markedly decreased after the combination of the inhibitors of the complex I and III of ETC–Rotenone and Antimycin A was applied to the cells (marked as Rotenone in Figure 4a), indicating that the complex I and/or III are crucial for OXPHOS in *Spalax* cells.

## 3. Discussion

Over the last decade, targeted metabolomics has been a method of choice for the studies of specific metabolic phenotypes in mammals [23], microbial [24], and cancer cells [21,25]. In the current study, the metabolic flux analysis of ^13^C_6_-Glc was used as a targeted-metabolomics tool for a comprehensive exploration of glucose metabolism in primary *Spalax* and rat skin cells under ~20% (normoxia) and 1% O_2_ (hypoxia) atmosphere. The specific labeling pattern that appears after supplying the cells with ^13^C_6_-labeled Glc is a reliable framework to determine the intracellular fluxes of Glc-derived carbons. Cells cultured with a stable isotope-labeled Glc consume and metabolize it, allowing heavy glucose-derived carbons to be integrated into metabolic reactions and appear as heavy metabolites.

When facing low oxygen supply, mammalian cells respond similarly, via the stabilization of HIF-1α, the master regulator of the cellular response to hypoxia, firstly discovered in 1995 by G. Semenza and G. Wang [26]. HIF induces a specific genetic switch to hypoxic metabolic phenotype that facilitates the adaptation and survival of cells under hypoxia [27]. HIF-1 is described as a heterodimer consisting of two subunits HIF-1α and HIF-1β [26]. The regulatory activity of HIF proteins is determined by the stability of the HIF-1α subunit. Under normoxic conditions, HIF-1α is constitutively degraded, whereas under low oxygen, it stabilizes and translocates into the nucleus where it orchestrates the gene expression [28]. Thus, the lowering of oxygen concentrations is accurately sensed by HIF-1α which orchestrates cellular homeostasis under hypoxia. Nonetheless, what are the consequences for the metabolic phenotype when HIF-1α remains stabilized also under normoxia? To date, HIF-1α over-expression is reported in various cancer lines, where it facilitates metastasis, tumor progression, and resistance to anticancer drugs [29,30]; however, to our knowledge, there is almost no data about the metabolic phenotype of ‘HIF-1α-positive’ mammals. The present results confirm that normoxic *Spalax* cells express a higher level of HIF-1α compared to the rat cells, and are further upregulated under low oxygen, which is in line with the previously reported in-vivo experiments on *Spalax* and rat kidney mRNA expression [31]. Consequently, this suggests that the HIF-1α mastered the metabolic phenotype also in normoxic *Spalax* cells. In particular, this is evidenced by the upregulation of anaerobic glycolysis [32], glycosylation [33], and uncoupling of the glycolysis and the TCA cycle [34]. Higher Lact production, active cytosolic Glc homeostasis, and decreased PDH rate in *Spalax* compared to the rat were evident in normoxic *Spalax* cells after 24 h. Along with the indicated canonical signatures of HIF-1α in resting *Spalax* cells, it did not demonstrate upregulated Glc consumption that is typically attributed to its action. Thus, the state of oxygen-independent HIF-1α stabilization (named ‘pseudohypoxia’; reviewed by Philip S Macklin [35], and S. Mohlin [36]) was indeed observed in normoxic *Spalax* skin cells as well as the HIF-1α overexpression previously observed in the liver transcriptome (Figure 4c–e). Apparently, the ‘pseudohypoxia’ in *Spalax* cells correlates with the semi-independent simultaneous functioning of both TCA cycle and glycolysis. 

The tracing of labelled Glc indicates HIF-1α-controlled specific metabolic events in *Spalax* cells under normoxia, such as upregulated glycolysis, and at the same time, the prevention of Glc-derived pyruvate entry to the TCA cycle due to the decrease of PDH activity [34]. This metabolic state can be defined as anaerobic glycolysis. The decreased PDH rate indicated in normoxic *Spalax* cells interrupts Please ensure intended meaning is retained further metabolism of Pyr M + 3 in the TCA cycle. Uncoupling the glycolytic carbons flux from the TCA cycle reactions can cause an NAD^+^ shortage that is ultimately needed for trioses metabolism by glyceraldehyde dehydrogenase. Pyruvate mainly fuels the TCA cycle or is converted by ALT to Ala, or by LDH to Lact [17]. Resting *Spalax* cells produce and secrete more Lact M + 3 than rat cells, therefore maintaining the NAD^+^ pool and rejecting the excess of carbons from the cells following HIF-1α guidance. The decreased Ala levels reported in *Spalax* cells as compared to rat cells can be explained by an αKG shortage due to the diminished pyruvate flow to the TCA cycle where αKG is produced and is required for the ALT reaction. Thus, normoxic *Spalax* cells can maintain glycolytic machinery in semi-independent mode from the TCA cycle. There are two possible metabolic scenarios for glucose carbons in this case: to be exported from the cell, or circulate between PPP and the glycolytic axis, and finally, be distributed between anabolic endpoints. Both cases are detected in normoxic *Spalax* cells in the study. The extracellular Lact M + 3 concentrations (indicating rejection of Glc-derived carbons from the cell) are significantly higher for *Spalax* cells compared to those of the rat, as well as the upregulated PPP and the distribution of heavy Glc-derived carbons between specific anabolic end-points such as UDP-GlcNAc and GSH that were pronounced in *Spalax* cells. 

The labeled Glc carbons which were detected in the structures of UDP-GlcNAc M + 6 and its precursord-GlcNAc M + 6 are sourced from the heavy F6P M + 6 directed to HBP. Levels of specific MIs of d-GlcNAc and UDP-GlcNAc may shed light on the shuttling of Glc-derived carbons between the main glycolysis, PPP, and the TCA cycle [37]. The incorporation of the TCA cycle-derived heavy Ac-CoA M + 2; +1; +0 into the d-GlcNAc (detected as d-GlcNAc M + 8;7;6), PPP-derived labeled UTP M + 5, and non-labeled UTP M + 0 ribose sugar into UDP-GlcNAc (detected as UDP-GlcNAc M + 13; +12; +11; +8) was traced. Thus, UDP-GlcNAc harbors Glc carbons diverted from the main glycolysis, PPP, and the TCA cycle. The domination of UDP-GlcNAc M + 13; +8 MIs detected in both normoxic and hypoxic *Spalax* cells (Figure S1d) is the result of a powerful Glc flux to HBP. In contrast with published data [38] and with our initial assumption that hyaluronic acid production stands behind the viscous medium, *Spalax* skin fibroblasts did not direct glucose to HA (at least under normoxia). The only way for glucose in HBP besides HA is UDP-GlcNAc production with the subsequent glycosylation of proteins [14]. The upregulated UDP-GlcNAc production and the dramatic response on inhibition of GlcNAc phosphotransferase suggest a particular importance of glycosylation for the homeostasis of *Spalax* cells. 

The time-resolved enrichment of the metabolic landscape under normoxic conditions with heavy nutrients indicates GSH as one of the metabolites harboring both Glc-derived. The appearance of appropriate heavy GSH M + 1; +2; +3; +4, as well as the sum of all detected MI of GSH (Figure S2B) are dominant in *Spalax* cells. These pronounced fluxes suggest a specific metabolic importance of GSH for the maintenance of the *Spalax* metabolic pattern, since the elimination of reactive oxygen species (ROS) is vital for cells under hypoxic conditions. Hypoxia, and especially periodic sharp changes from hypoxia to oxygenation which are likely to occur in the *Spalax* underground environment, are well described as inducers of ROS production [39,40]. GSH is well known as an abundant intracellular antioxidant [41] as well as a cofactor of GSH-peroxidase; it participates in a non-specific reduction of hydroperoxides resulting in the formation of its oxidized form GSSG. Thus, GSSG levels indirectly indicate the intensity of the intracellular free radical processes. The balance between reduced and oxidized forms of intracellular glutathione reflects the ability of cells to eliminate hydroperoxides. The GSH pool in *Spalax* cells is significantly more reduced than in rat cells and has the appropriate potential to cope with oxidative stress. Thus, *Spalax* cells maintain a massive intracellular GSH pool to prevent oxidative damage. 

The PDH is a primary source of Ac-CoA in the cell. Newly produced Ac-CoA M + 2 can be utilized in several ways: condensation with OAA M + 0 into Citr M + 2 to ‘feed’ the TCA cycle; withdrawal to the cytosol and processing into the de-novo synthesized fatty acids, or participate in the UDP-GlncNAc biosynthesis; otherwise, it can be used for acetylation processes (recorded in our study as acetyl carnitine (ALCAR) and acetyl-derivate of some amino acids (e.g., Gln and Asp). The obtained results suggest the prevalence of two scenarios in normoxic *Spalax* cells as compared to the rat, namely shuttling of produced heavy Ac-CoA M + 2 to HBP (detected as UDP-GlncNAc M + 8) that was discussed above, and its buffering specifically as an ALCAR M + 2. Hypothetically, maintaining higher levels of ALCAR in *Spalax* cells suggest an alternative storage of highly energetic, easily available acetyl groups. However, this hypothesis requires further extensive study. 

As mentioned above, the PDH rate for *Spalax* cells is significantly lower than that in rat cells due to HIF-1α regulations. Apparently, the pyruvate anaplerosis via pyruvate carboxylase is not mastered by HIF-1α, and can serve as an alternative strategy to maintain the TCA cycle machinery by filling it up with carbons under decreased PDC function. PC-metabolized Pyr M + 3 in *Spalax* cells as OAA M + 3 flux followed the TCA cycle reactions and unequally divided at the αKG step between further cycle’s metabolism (lesser part, while in rat cells the TCA cycle metabolism is major for PC-derived M + 3 metabolites) and the path to collagen biosynthesis. The enhanced cataplerosis of Glc-derived carbons from the TCA cycle to collagen production in *Spalax* cells is evidenced by the appearance of higher levels of HPro M + 5 as compared to levels in the rat cells. Apparently, the HPro M + 5 is a Citr M + 5 that had been metabolized to αKG M + 5 and further to Pro M + 5, which was finally incorporated in collagen and undergone hydroxylation by prolyl oxidase [42]. Thus, normoxic *Spalax* cells more actively use pyruvate anaplerosis than the rat cells, but at the αKG step, divert the carbons’ flux from the downstream TCA cycle reactions to collagen biosynthesis. Moreover, a substantial part of Glc-originated carbons entered the mitochondria and most of the newly consumed Gln in normoxic *Spalax* cells were diverted to the metabolism along with the axis Glu→Pro→HPro instead of processing in the TCA cycle reactions.

The majority of carbons entered the TCA cycle via PDH as Ac-CoA M + 2 and converted to CitrM + 2; however, they were not processed via the downstream cycle’s reactions in normoxic *Spalax* cells, which was evidenced by lower levels of the Citr M + 2; αKG M + 2; Succ M + 2; Mal M + 2 (Figure 3a–d) when compared to the rat cells. These carbons were rejected from the central metabolism in favor of collagen production (measured as HPro M + 2) (Figure 3f). Indeed, most of all detected heavy Glc-carbons metabolized in the mitochondria of *Spalax* cells (appeared as M + 1, +2; +3; +4; +5) were finally observed as MI of HPro, a product of collagen degradation (Figure S4). 

Under normoxia, the levels of HIF-1α decreases via proteasome degradation. The process of degradation of HIF-1α is initiated by αKG-dependent prolyl hydroxylase (PHD) via the hydroxylation of its Pro residues [43]. αKG is a substrate for PHD [44] and 2HG, being the enantiomer of aKG, competes with it for binding to PHD, and may inhibit its function, and subsequently prevents HIF-1α degradation [45,46]. Although the role of 2HG in the inhibition/activation of PHD is controversial [47,48], our data suggest a possible involvement of 2HG in PHD inhibition. Theoretically, the higher HIF-1α levels observed in normoxic *Spalax* cells maintains at the account of upregulated PC driven flow of Glc to 2HG production (detected as 2HG M + 3). The hypoxic metabolic landscape of *Spalax* cells is characterized by both decreased levels of total αKG (sum of areas of all αKG MI’s Figure S5(Bb)) and upregulation of 2HG production as a result of tunneling of Glc (2HG M + 2; +3; +4) (Figure 3i and Figure S2(Ai–Ak)). Both decreased levels of the essential participant of the PHD reaction αKG and upregulated production of 2HG apparently lead to the stabilization of HIF-1α and its steeper increase in hypoxic *Spalax* cells compared to rat cells. These suggestions agree with the previously reported higher levels of HIF-1α and its target (Epo) in the *Spalax* kidney in vivo compared to the rat kidney under hypoxia [31]. This suggests a reciprocal regulation of HIF-1α levels and metabolism in *Spalax* cells. Thus, rewiring of Glc in hypoxic *Spalax* cells is involved in the development of the hypoxic HIF-1α mastered phenotype as an additional factor of HIF-1α stabilization in a reciprocal regulation mode. 

The ‘true’ hypoxia caused HIF-1α induction in cells of both species, while maintaining the gap observed under normoxia (levels HIF-1α in the *Spalax* were higher compared to the rat (Figure 4). Hypoxic Glc homeostasis in the cytosol of both cell types seems to be ‘canonically’ mastered by HIF-1α. The upregulation of Glc consumption, the glycolytic flow, and lactate secretion are classical metabolic signatures of the anaerobic cell bioenergetics mode. While in rat cells, a clear reprograming to hypoxic mode was observed, *Spalax* cells exploit a metabolic pattern generally similar to normoxia. Most of the consumed Glc carbons in *Spalax* cells are harbored as glycerol (data not presented here), GSH, UDP-GlcNAc, or secreted outside as Lact and Ala. Similar to normoxia, the TCA cycle of *Spalax* cells functions in a ‘semi-independent’ manner due to a low PDH rate that does not change in *Spalax* cells under hypoxia as compared to the normoxia condition, while in the rat, the PDH rate increased in comparison to both normoxic rat and hypoxic *Spalax* cells. In hypoxic *Spalax* cells, the TCA-cycled Glc-derived carbons were finally distributed between 2HG, HPro, and Asn, levels of which were increased compared to those under normoxia, probably on the account of the decreased Glu secretion. Notably, the upregulated de-novo production of 2HG, Pro, HPro, and Asn acts as a specific signature of hypoxic *Spalax* cells compared to the rat cells. Apparently, *Spalax* cells use this metabolic frame regardless of the availability of oxygen; however, a hypoxic response was clearly detected as the upregulation of these metabolites. Although the hypoxic rat cells showed a similar induction of 2HG, Pro, HPro, and Asn biosynthesis, it was much weaker when compared to *Spalax* cells.

The pronounced upregulation of de novo Asn production observed in both resting and exclusively in hypoxic *Spalax* cells is probably involved in the protein modification that occurs via N-glycosylation of the side-chain amide nitrogen of Asn residues by N-GlcNAc [49] that was uniquely upregulated in *Spalax* cells. N-glycosylation is involved in the control of proteins folding that occurs in the endoplasmic reticulum (ER) and is highly sensitive to oxygen availability [50]. The increased levels of HIF-1α and all measured participants of glycosylation (UDP-GlcNAc and Asn) in normoxic *Spalax* cells, and their synchronic upregulation under hypoxia are easily traced and can be described as specific reciprocal interactions of HIF-1α [33] and glycosylation that influence HIF-1α stabilization [51]. This suggests that the improved glycosylation in *Spalax* cells serves as a specific adaptation to maintain an appropriate signaling and protein folding in ER under hypoxia. This issue is, however, a subject for further investigations.

Hypoxic *Spalax* cells are characterized by significantly upregulated levels of HIF-1α as compared to both its normoxic values and to hypoxic rat cells. High HIF-1α, with a simultaneous increased production of 2HG, suggests a specific role of 2HG as the factor that prevents HIF-1α degradation even under regular O_2_ atmosphere. The upregulation of 2HG production observed in hypoxic *Spalax* cells compared to normoxia supports the suggestion that levels of HIF-1α can be controlled not only by oxygen concentrations, but also by levels of 2HG (branching of the carbon flux from TCA cycle to 2HG in this case). It was markedly indicated that much more glucose carbons are detected in 2HG under hypoxia. Nearly all metabolic pathways for Glc (M + 2; +3; +4) were diverted to 2HG biosynthesis under hypoxia.

2HG can be easily returned back to the TCA cycle metabolism as αKG through the action of the mitochondrial d-2-hydroxyglutarate dehydrogenase (d-2HGDH) [52]. The 2HG oxidation uses a specific machinery containing FAD and an electron acceptor that was identified by Struys et al. as membrane-associated electron-transfer flavoprotein ubiquinone oxidoreductase (ETFQO) [53] to pass electrons to ETC [54,55]. A supportive evidence comes from an earlier-published transcriptome [56] showing that the liver expression of both 2HGDH and ETFQO is much higher in *Spalax* as compared to the rat (Figure S5(Bm,Bn)), all in agreement with the idea suggested here about the circulation of Glc carbons between αKG and 2HG. Another implication of the raised 2HG production that requires further investigation is the specific epigenetic landscape in *Spalax* cells. The inhibition of αKG-dependent oxidases causes alterations in hydroxylation, demethylation, halogenation, desaturation, epoxidation, ring formations, and other reactions [57]. This ultimately leads to specific changes in the epigenetic landscape and intracellular homeostasis.

Hypoxia promotes Pro and HPro production in tumor cell lines, which is a part of the hypoxia response through the regulation of HIF-1α [58]. A remarkable observation is the similar hypoxia-induced upregulation of HPro levels indicated in *Spalax* cells. Free HPro is not proteinogenic amino acid [59], and its degradation utilizes the same enzyme machinery as for Pro [6], as it was described above. Therefore, hypoxia-induced upregulation of both free Pro and HPro levels suggests an activation of the Pro shuttle machinery. This is partially evidenced by liver transcriptome data on *Spalax* and rat (Figure S5(Bd,Be)). Thus, Pro and HPro can be considered as an alternative bioenergetics substrate in *Spalax* cells for survival under hypoxic stress.

Hypoxia, and especially sharply changing hypoxia and re-oxygenation states, cause an imbalance between the production and elimination ROS in the cell. Although higher levels of GSH production were observed in *Spalax* cells under normoxia, low oxygen conditions caused further significant amplification of carbon fluxes to GSH biosynthesis in *Spalax* cells (Figure S2B). Glutathione peroxidase reaction which utilizes GSH to eliminate hydroperoxides requires NADPH, which is in a good agreement with higher levels of NADPH observed in *Spalax* cells under both normoxia and hypoxia. An indirect evidence of upregulated ROS-induced processes under hypoxia is the increased levels of GSSG that were observed in both species, while GSSG levels in rat cells were higher as compared to *Spalax* cells. The two evidences introduce the *Spalax* metabolic pattern as a system that produces fewer disturbances in ROS production in the situations of changing normoxic and hypoxic states, and/or massive GSH biosynthesis that prevents intracellular injuries caused by ROS. The last suggestion is supported by data on the antioxidant system in the Harderian gland of *Spalax* published by B. Caballero et al. [6] . According to the published data, *Spalax* exhibits remarkably low bimolecular damage in the oxidative stress situation as compared to the Syrian hamster, because of a stronger enzymatic antioxidant system such as superoxide dismutase, catalase, and glutathione reductase that reduces GSSG to GSH.

## 4. Methods

### 4.1. General Experimental Design

The work was carried out on a cell culture model and on cells/tissues taken from wild mole rats and laboratory rats. For metabolic studies, cells were treated by hypoxia (1% O_2_) and compared to normoxic cells. Extracts from cultured cells were subjected to SDS-PAGE electrophoresis for protein analyses by western blot using specific antibodies.

### 4.2. Animals

For the experiments three newborn blind-mole rats (*Spalax erenberghi*) and three newborn rats (*Rattus norvegicus*) were used. *Spalax* individuals were captured during several field expeditions (in 2018, 2019 around Carmel mt. area) from the underground nests during the procreation period and subjected to the procedure immediately upon arrival to the lab (no more than 2 h after capturing). Thus, they represent a metabolic pattern of wild animals and feature with specific characteristics such as hypoxia [31] and cancer [7] tolerance. Pregnant rats were obtained from Envigo (Jerusalem, Israel) and caged under ambient conditions in the Animal Facility of the Institute of Evolution, University of Haifa, until the birth of the pup rats (in 2013, 2019). Therefore, the experimental animals represent two different metabolic patterns: hypoxia-tolerant (*Spalax*) and hypoxia-sensitive (rat). The newborn *Spalax* and rats were anaesthetized with Isoflurane overdose and subjected to the primary cells isolation protocol described below. All manipulations with animals were approved by the Institutional Ethics Committee (Reference #316/14, 420/16 and 671/19).

### 4.3. Cell Culture

Primary skin cells were isolated from the animals’ underarm skin region according to the method proposed by S. Glaysher and I. A. Cree [60]. The primary cells were cultured in DMEM-F12 medium (Biological Industries, Beit Haemeq, Israel) supplemented with 5% fetal bovine serum (FBS) under ambient humidified atmosphere (5% of CO_2_ and 95% of air) at 37 °C, and after the second passage cells were split into six-well plates (5 × 10^5^ cells/well) for the targeted metabolomics experiment (for six technical replicates) ; into the Seahorse XF96 (Agilent Technologies, Santa Clara, CA, USA) cell culture plate (2 × 10^4^ cells/well) for the live-cells metabolic assays, and into 100 mm Corning^®^ (Tewksbury, MA, USA) culture dishes (1 × 10^6^ cells/dish) for the Western blot and hyaluronic acid assays.

### 4.4. Experiment Schedule, Extraction, and LC-MS Analysis

The cells were divided into groups (six replicates each) and incubated for 12 h in 5% of CO_2_ and 95% of air at 37 °C in DMEM-F12 medium with FBS. Further, the culture medium was removed and replaced for the next 12 h with more ‘physiological’ custom freshly-prepared serum-free medium (SFM). SFM consists of Dubellco’s Modified Eagle Medium-F12 glucose and Gln free (Gibco, Thermo Fisher Scientific, Waltham, MA, USA) supplemented with bovine serum albumin/insulin/transferrin (Insulin-Trans-Sel-X, Gibco); 2.5 mg/L of ascorbic acid phosphate; 1 mg/L of glutathione; 0.0003 mg/L of ammonium metavanadate; 0.25 nM of manganous chloride; 0.1 mM of acetate; 5 mMg of glucose; 0.6 mM of Gln.

### 4.5. ^13^C_6_ Glucose Tracing Experiment Design

Two hours prior to the beginning of the experiment, a fresh medium with labeled ^13^C_6_ glucose was prepared based on SFM described above with the non-labelled glucose replaced by 5 mM of ^13^C_6_ glucose (U-13C6, 99%, Cambridge Isotope Laboratories, Tewksbury, MA, USA, CLM13961), as well as PBS as control. Containers with opened lids were placed into the chamber with 1% of O_2_ atmosphere for deoxygenation. The cells were transferred to the hypoxic chamber, medium removed, and after PBS washing, replaced with deoxygenated medium containing ^13^C_6_ glucose. The same manipulations were done for the normoxic conditions, but the medium and PBS together with the cells were placed into the regular CO_2_ incubator (21% O_2_). The rest of the fresh labeled medium (three aliquots of 2 mL each) was kept together with normoxic and hypoxic cells during the study.

The cells supplied with labelled ^13^C_6_ glucose were incubated for 24 h under normoxia (~20% of O_2_) in a regular CO_2_ incubator, or hypoxia (~1% of O_2_) in the hypoxic chamber (HypOxystation^®^ H35, HypOxygen, Frederick, MD, USA). After the incubation, the culture medium was harvested, cells were washed twice with ice-cold PBS, and intracellular metabolites were extracted using methanol/acetonitrile/water solution (5:3:2) (Mobile phase) on a rocker shaker (10 min at 4 °C). The extracts and medium samples were centrifuged (16,000× *g* 10 min, 4 °C), the supernatants (medium samples after 1:50 dilution with mobile phase) were transferred to −80 °C and stored until subjected to LC-MS analysis.

### 4.6. LC-MS Metabolomics Analysis

LC-MS metabolomics analysis was performed as described previously [61]. Briefly, Thermo Ultimate 3000 high-performance liquid chromatography (HPLC) system coupled to Q- Exactive Orbitrap Mass Spectrometer (Thermo Fisher Scientific) was used with a resolution of 35,000 at 200 mass/charge ratio (*m*/*z*), electrospray ionization, and polarity switching mode to enable both positive and negative ions across a mass range of 67 to 1000 *m*/*z*. HPLC setup consisted ZIC-pHILIC column (SeQuant; 150 mm × 2.1 mm, 5 μm; Merck, MA, USA), with a ZIC-pHILIC guard column (SeQuant; 20 mm × 2.1 mm). 5 µL of biological extracts were injected and the compounds were separated with a mobile phase gradient of 15 min, starting at 20% aqueous (20 mM ammonium carbonate adjusted to pH 2 with 0.1% of 25% ammonium hydroxide) and 80% organic (acetonitrile) and terminated with 20% acetonitrile. Flow rate and column temperature were maintained at 0.2 mL/min and 45 °C respectively, for a total run time of 27 min. All metabolites were detected using mass accuracy below 5 ppm. Thermo Xcalibur was used for data acquisition, TraceFinder 4.1 was used for data analysis. Peak areas of metabolites were determined by using the exact mass of the singly charged ions. The retention time of identified metabolites was predetermined on the pHILIC column by analyzing an in-house mass spectrometry metabolite library that was built by running commercially available standards. Each metabolite peak area value analyzed in the sample, was normalized to protein amount. Protein concentration was measured by Pierce™ Modified Lowry Protein Assay Kit (Thermo Fisher Scientific).

### 4.7. Oxygen Consumption Rate (OCR) and Extracellular Acidification Rate (ECAR) Measurements

The bioenergetics machinery of the primary *Spalax* and rat cells (two lines of each species) was explored by challenging them with the Mito and Glyco stress test assays using the Seahorse XF96 analyzer (Agilent Technologies).

Cells were seeded on XFe96 well plates and incubated at 37 °C, 5% CO_2_ for 24 h. The medium was then replaced with 180 μL of unbuffered assay media [Sigma D5030, (Merck)] supplemented with 10 mM glucose, 1 mM pyruvate and 2 mM Glutamine (pH 7.4) for Mitochondrial Stress Test or 2 mM Glutamine only for Glycolysis stress test. Cells were then placed at 37 °C in a CO_2_-free incubator for 1 h. During the experiment, 1μM oligomycin A (Sigma), 1.0 μM FCCP (Sigma) and 50 μM rotenone and antimycin A mixture (Sigma) were injected sequentially. For Glycolysis Stress test, the assay medium was supplemented with 2 mM glutamine. The cells were deprived of glucose for 1 h. During the experiment, 10 mM glucose (Sigma), 1 μM oligomycin A (sigma) and 50 mM of 2-Deoxyglucose (Sigma) were injected sequentially. OCR and ECAR were normalized to the protein content in each well calculated at the end of the experiments by using the Pierce™ Modified Lowry Protein Assay Kit (Thermo Fisher Scientific). Data were analyzed using Wave software version 2.6. 

### 4.8. Hyaluronic Acid Assay

The Purple–Jelley HA assay (Biocolor, Carrickfergus, UK) was used for quantification hyaluronic acid (HA) content in the primary skin cells and tissues. Skin tissues were sampled from skin tissues stored at −80 °C. Tissues were previously harvested from normoxic rats and *Spalax* (three adult animals of each specie) [62], and HA was extracted and analyzed according to the protocol provided in the commercial kit for HA extraction and quantification. Since the protocol provided with the kit does not describe the procedure of HA extraction from cells, an appropriate method was proposed. Briefly, three dishes (semi-confluent) of each cell type (see the ‘Cell culture’ section) were used for analysis. The cells were lysed (three freeze-thaw cycles with liquid N_2_) immediately after culture medium removal and two PBS washes. Cells were harvested with rubber scrapper into vials (lysates from three dishes pooled in the one vial), dried until semi-dry condition (Eppendorf Concentrator 5301, Hamburg, Germany), and about 100 mg of the semi-dry cell lysates were processed together with the tissues extracts as described in the kit’s protocol. The HA concentration were calculated based on calibration curve with standard provided in the kit and presented as µg/g.

### 4.9. Western Blot

The primary *Spalax* and rat cells (three lines of each species) were exposed to 24 h of normoxia or hypoxia simultaneously with the cells for targeted metabolomics. The cells were lysed by RIPA-buffer on ice-bath immediately after the experiment. The lysates (40 µg protein) were subjected to 10% SDS-PAGE and transferred to a nitrocellulose membrane. For the detection of HIF-1α protein, the anti HIF-1α (28b):sc-13515 (Santa Cruz Biotecnology, Dallas, TX, USA) was used as primary, and anti-mouse IgG, HRP-linked#7076 (Cell Signaling, Danvers, MA, USA), as secondary antibody. The signal intensity was normalized by tubulin and actin probed with anti-Tubulin (10D8; sc-53646) and anti-Actin (I-19; sc-1616) antibodies.

### 4.10. Statistical Analysis

Raw data were normalized and *p* values were calculated with the GraphPad Prizm v. 8 software using unpaired Student’s *t*-test (*p* < 0.05 was considered to be statistically significant as specified in the figure legends).

## 5. Conclusions

We suggest the following glucose distribution for *Spalax* cells. Glucose homeostasis in the primary *Spalax* skin cells is mastered by upregulated HIF-1α under both normoxia and hypoxia. Higher levels of HIF-1α are maintained due to the prevention of its degradation, because of the higher production of 2HG and upregulated glycosylation. Glucose tracing in *Spalax* cells characterizes a ‘semi-independent’ functioning of the glycolysis and the TCA cycle regardless of oxygen availability. The *Spalax* cytosolic glucose machinery does not depend upon TCA-derived NAD^+^. The shortage of NAD^+^ is covered by activated LDH reaction. In general, both normoxic and hypoxic *Spalax* cells employ anaerobic glycolysis. Glc carbons intensively circulate between PPP and the main glycolytic axis maintaining an appropriate pool of NADPH and finally harbor as GSH, glycerol, and UDP-GlcNAc. NADPH also acts as a bridge that links the cytosolic and the mitochondrial domains of Glc homeostasis. It is essential for both mitochondrial redox shuttles such as the Pro/HPro shuttle and production of d-2HG by IDH2. The mitochondrial metabolism of Glc-derived carbons is maintained mainly due to PC anaplerosis, while PDC flux stays low regardless of oxygen availability. *Spalax* cells utilize a specific cataplerotic pattern: glucose-originated carbons sidetrack from the TCA cycle reactions along the axis αKG→Glu→Pro→HPro and store as Pro/HPro in collagen structures outside the cell, or sink as Asn and further participate in the protein glycosylation. Collagen, in this case, can be considered a stress substrate that can be easily degraded by MPPs providing HPro/Pro for the Pro/HPro shuttle fueling. The redox shuttling of αKG-branched carbons is a specific metabolic signature of *Spalax* cells where it is employed for energy production and/or signaling via ROS. Hypoxic *Spalax* cells use a metabolic strategy similar to that under normoxia, but charged with a greater amount of Glc. The principal advantage of the Pro/HPro shuttle machinery is its independence of HIF-1α mastering. It does not shut down under hypoxia, neither causes lactic acidosis and apoptosis as it occurs in most eukaryotic cells. Thus, glucoses-derived metabolites in *Spalax* cells such as Pro, HPro, and 2HG can participate in bioenergetics processes and increase the effectiveness of the metabolic system. Massive collagen production requires more oxygen. Possibly, a higher oxygen consumption rate which was reported during the Seahorse experiment, can be described by non-respiration processes including the collagen production. Glucose metabolism maintains the antioxidant capacity of *Spalax* cells via a massive flux of carbons to GSH biosynthesis. Thus *Spalax* cells possess higher antioxidant potential compared to rat cells. The metabolic model for glucose homeostasis in *Spalax* cells presented here can serve as a solid platform for further studies on the strategies contributing to hypoxia tolerance, longevity, healthy aging, and cancer resistance of this mammal.

## Data Availability

Data is contained within the article and supplementary material.

## Supplementary Materials

The following are available online at https://www.mdpi.com/article/10.3390/metabo11110734/s1, Figure S1: Mass isotopologues distribution for selected metabolites in *Spalax* and rat cells, Figure S2: Selected characteristics of 13C6 glucose tracing under normoxia and hypoxia, Figure S3: Mitochondrial and TCA fluxes of Glc-originated carbons, Figure S4: The distribution of the metabolites along αKG-HPro axis, Figure S5: Glu-originated Asn, total αKG, and mRNA expression data.
