# Supplementary material for: Comprehensive Analysis of 13C6 Glucose Fate in the Hypoxia-Tolerant Blind Mole Rat Skin Fibroblasts
Source: Metabolites. 2021 Oct 27;11(11):734. doi: 10.3390/metabo11110734 (PMC8621580; doi:10.3390/metabo11110734)
Supplement: Supplementary file 1 [file metabolites-11-00734-s001.zip › metabolites-1432257-Supplementary.pdf]

# Supplementary Materials

## Figure S1

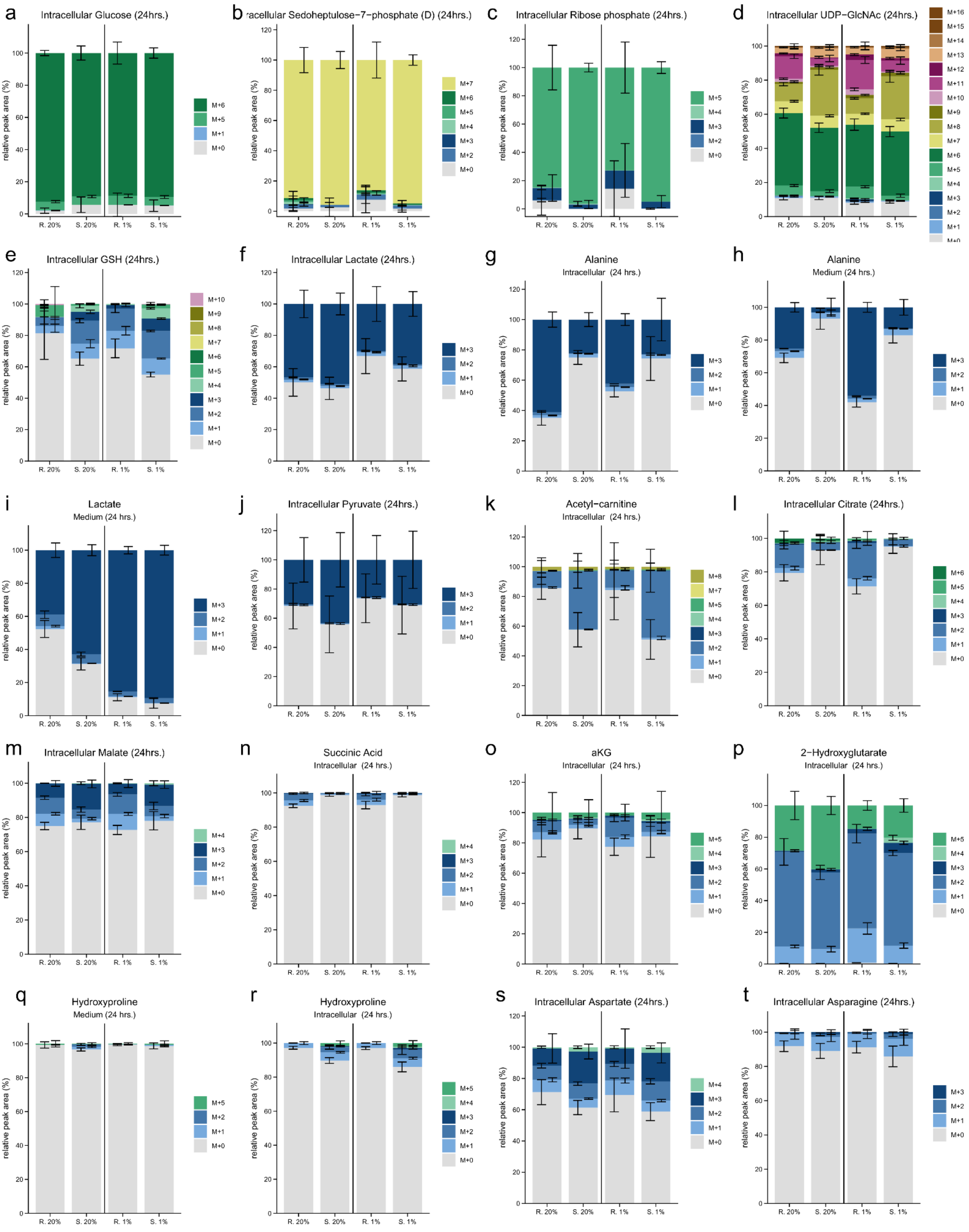


**Figure S1**. MID for selected metabolites (a-t) in *Spalax* and rat cells grown for 1 day in $^{13}C_6$ - glucose containing medium. S.20%, R. 20%, S.1%, R1% represent *Spalax* (S) and rat (R) cells exposed to an atmosphere containing 20% or 1% $O_2$, respectively. Error bars represent standard deviation of 6 or more biological repeats.

# Figure S2

## Panel A

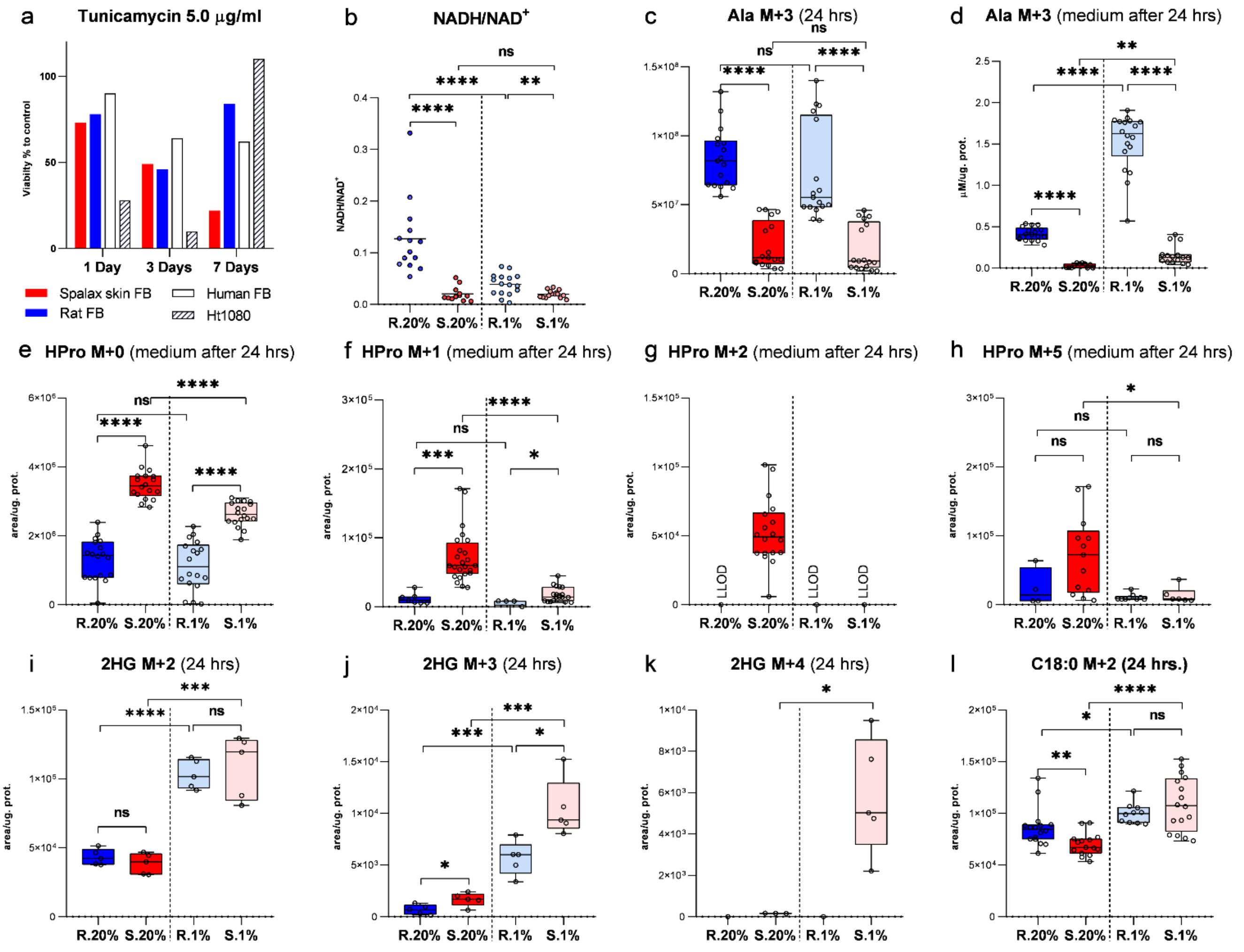


## Panel B

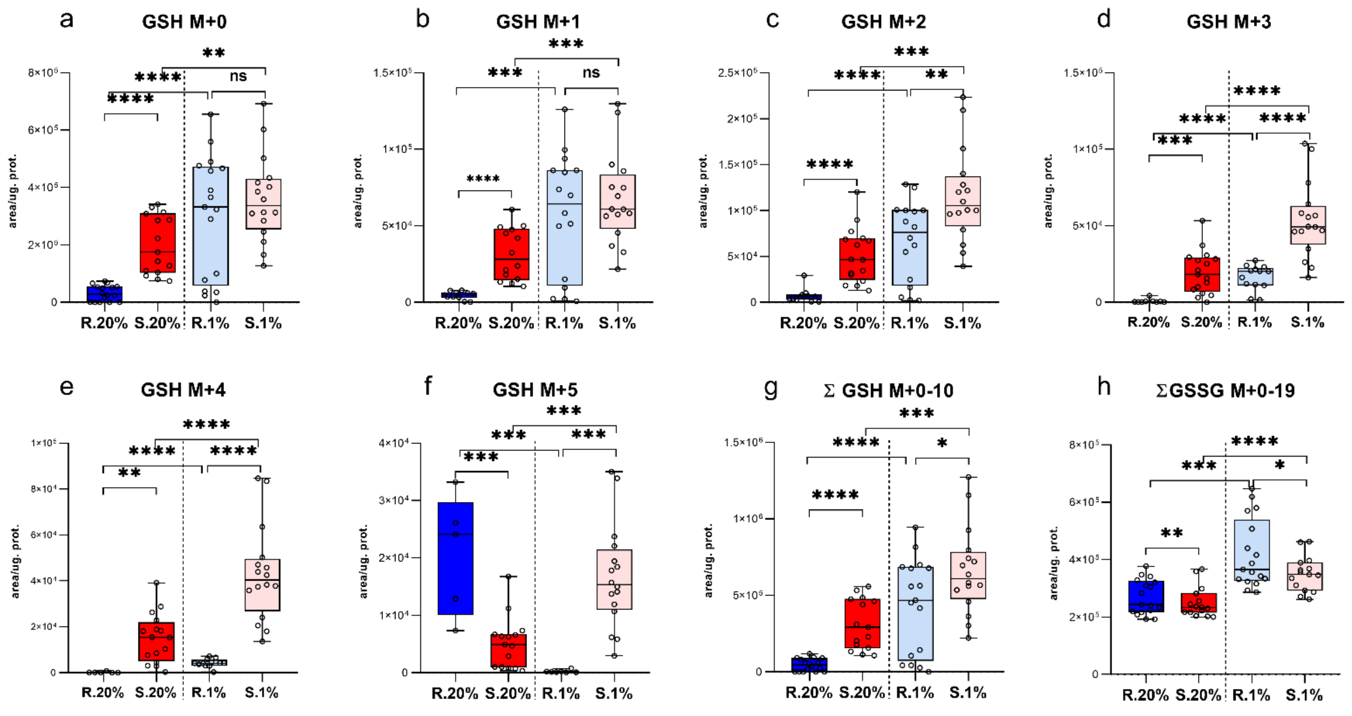


**Figure S2**. Panel A: Selected characteristics of $^{13}C_6$ tracing in *Spalax* and rat cells grown for 1 day in $^{13}C_6$ - glucose containing medium under normoxia and hypoxia. FB, fibroblasts; Ht1080, human fibrosarcoma cell line. Panel B: Mass isotopologues of GSH and sum of mass isotopologues of GSSG that contains Glc-derived carbons. S.20%, R. 20%, S.1%, R1% represent *Spalax* (S) and rat (R) cells exposed to an atmosphere containing 20% or 1% $O_2$, respectively; ns, (nonsignificant) p>0.05; *p<0.05; **p<0.01; ***p<0.001; **** p<0.0001, error bars represent standard deviation of 6 or more biological repeats. Every point on the chart represents one technical repeat.

# Figure S3

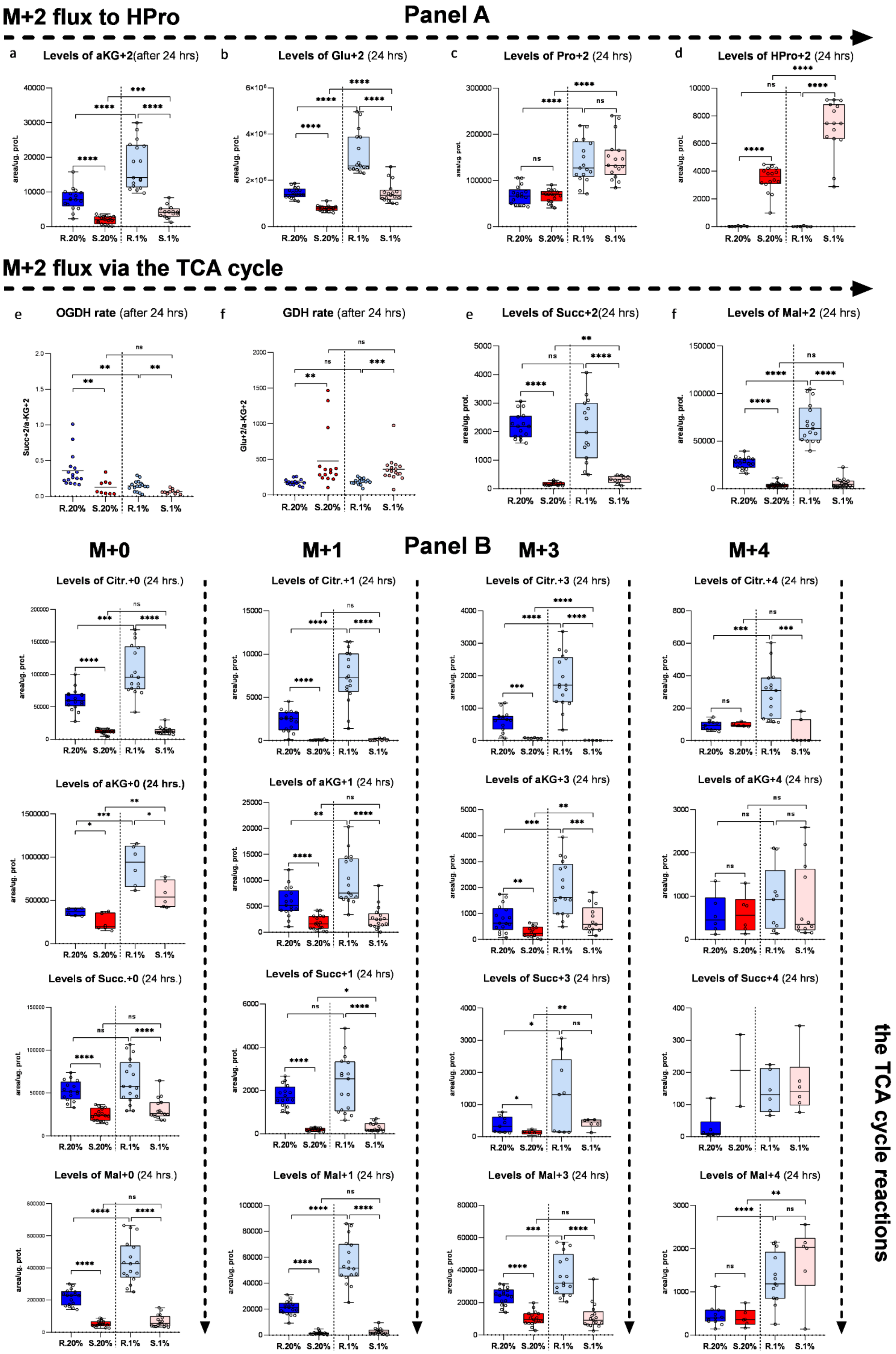


**Figure S3**. Selected mitochondrial fluxes of Glc-originated carbons in *Spalax* and rat skin cells grown for 1 day in $^{13}C_6$ - glucose containing medium under normoxia and hypoxia. Panel A:comparisons between M+2 fluxes to HPro and TCA. Panel B: TCA cycle reactions. S.20%, R. 20%, S.1%, R1% represent *Spalax* (S) and rat (R) cells exposed to an atmosphere containing 20% or 1% $O_2$, respectively; ns, (nonsignificant) p>0.05; *p<0.05; **p<0.01; ***p<0.001; **** p<0.0001, error bars represent standard deviation of 6 or more biological repeats. Every point on the chart represents one technical repeat.

# Figure S 4

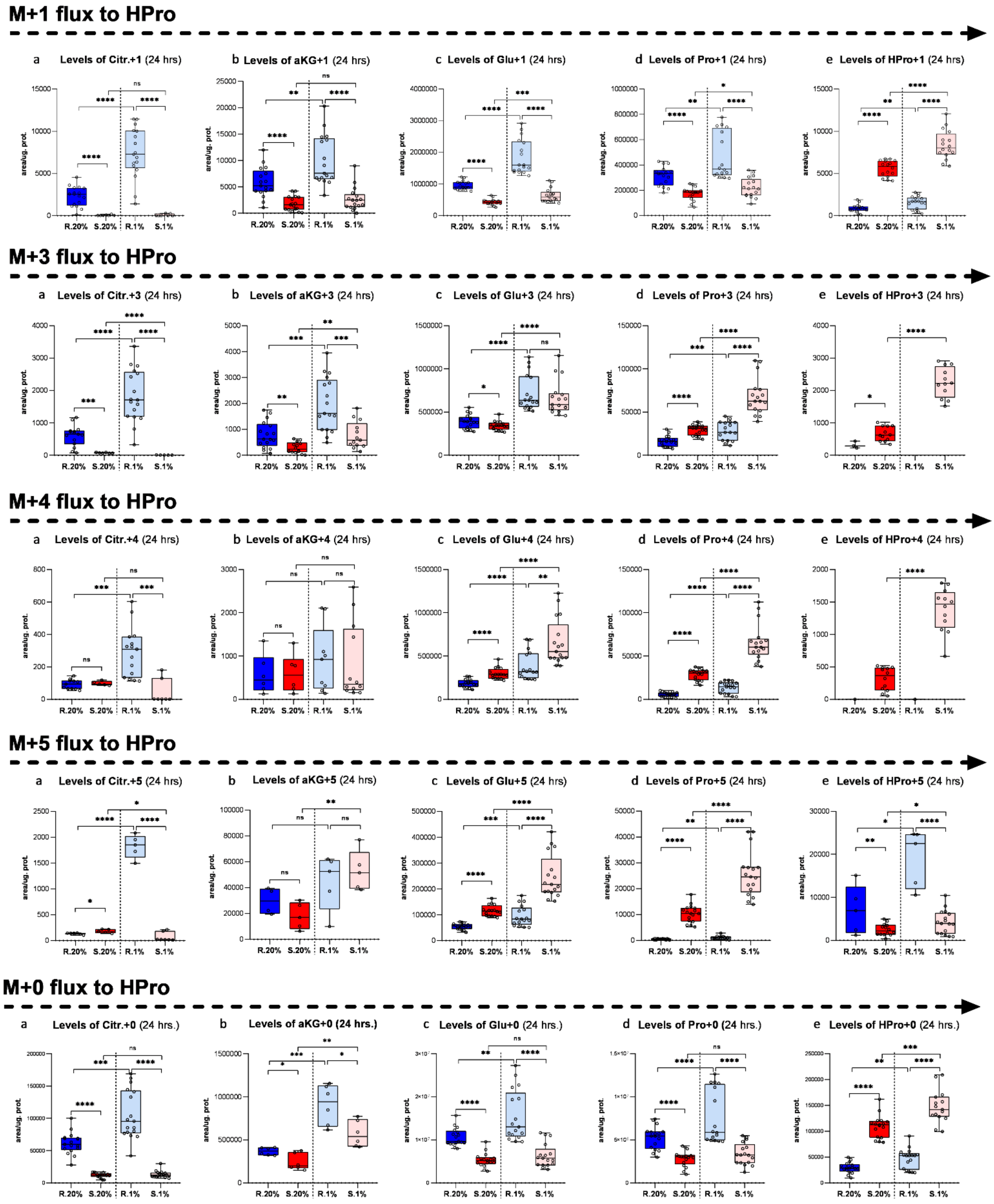


**Figure S4**. The distribution of measured metabolites along the axis Citr→αKG→Glu→Pro→HPro in *Spalax* cells as compared to the rat under normoxic and hypoxic conditions. S.20%, R. 20%, S.1%, R1% represent *Spalax* (S) and rat (R) cells exposed to an atmosphere containing 20% or 1% $O_2$, respectively; ns, (nonsignificant) $p>0.05$; *$p<0.05$; **$p<0.01$; ***$p<0.001$; **** $p<0.0001$, error bars represent standard deviation of 6 or more biological repeats. Every point on the chart represents one technical repeat.

# Figure S5

## Panel A

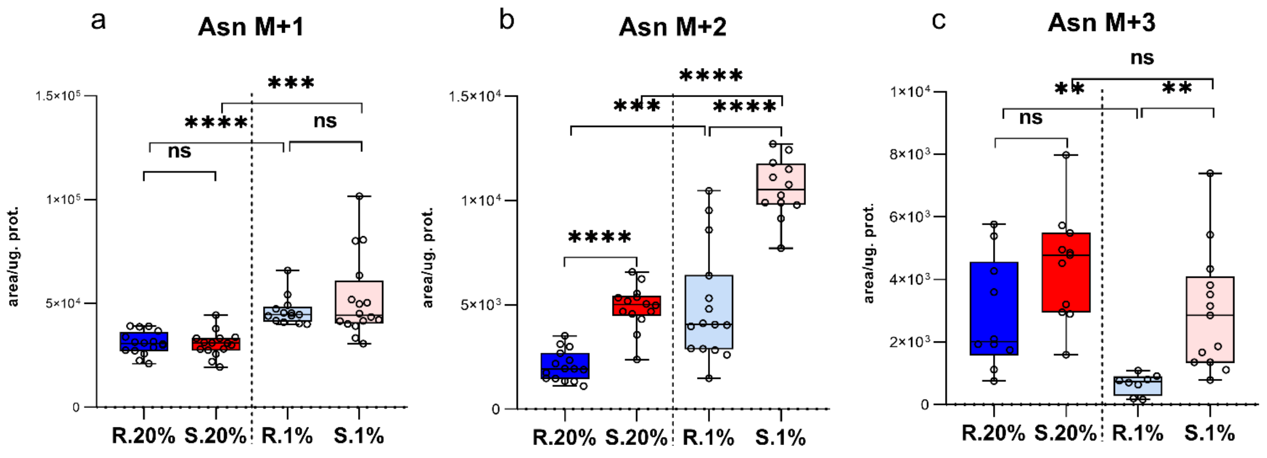


## Panel B

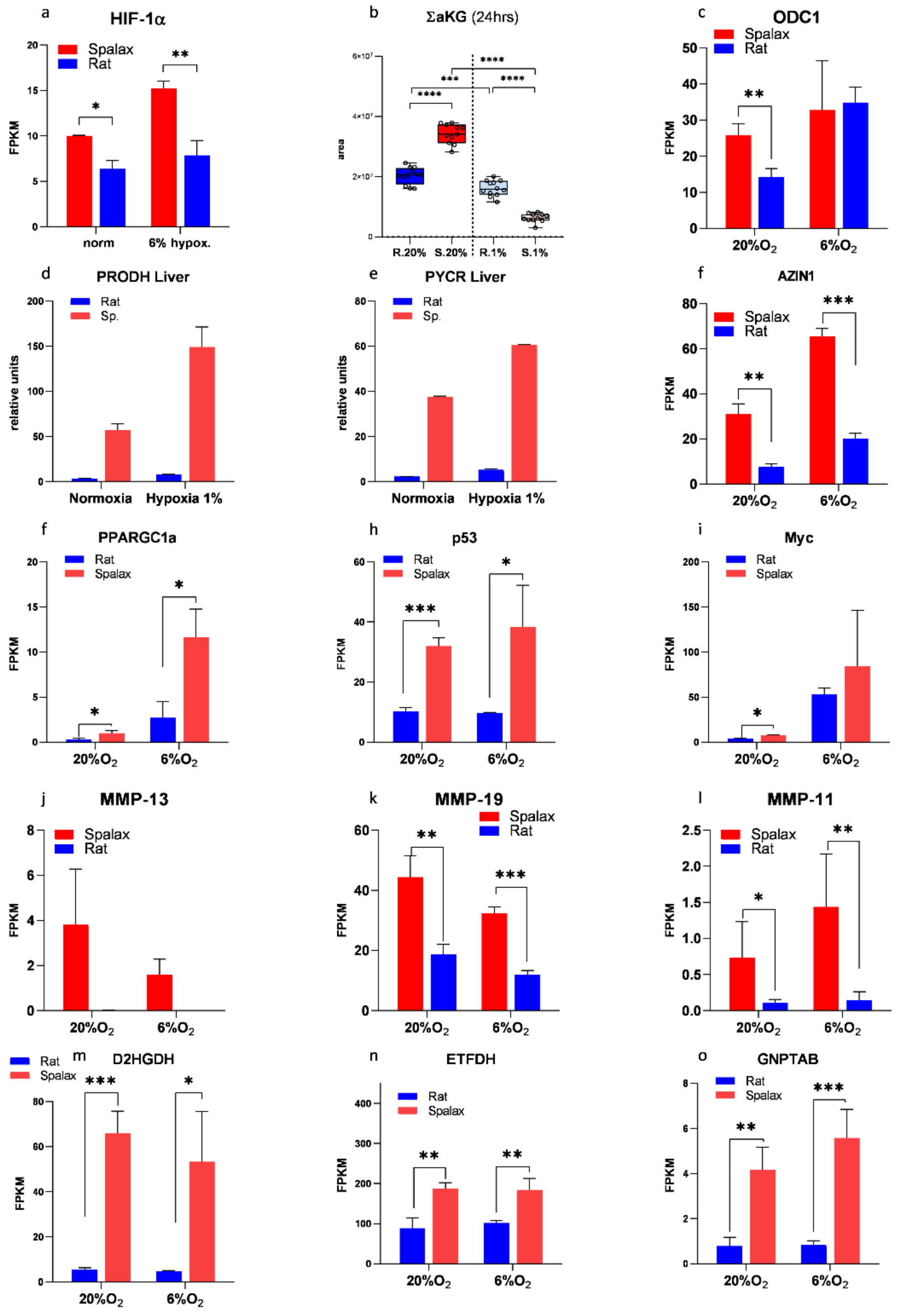


**Figure S5**. Panel A: Mass isotopologues of Asn containing Glc-derived carbons. . S.20%, R. 20%, S.1%, R1% represent *Spalax* (S) and rat (R) cells exposed to an atmosphere containing 20% or 1% $O_2$, respectively; ns, (nonsignificant) p>0.05; *p<0.05; **p<0.01; ***p<0.001; **** p<0.0001, error bars represent standard deviation of 6 or more biological repeats. Every point on the chart represents one technical repeat. Panel B: The levels of selected intracellular metabolites in *Spalax* and rat cells under normoxic and hypoxic conditions and mRNA expression of selected genes in *Spalax* liver tissues under normoxia and hypoxia (Retrieved from Reference # 62). FPKM, fragments Per Kilobase of transcript per Million mapped reads.
